# Functionalization of ZnO Nanorods with Au Nanodots via In Situ Reduction for High-Performance Detection of Ethyl Acetate

**DOI:** 10.3390/s24216931

**Published:** 2024-10-29

**Authors:** Qilin Wang, Wei Wang, Yizhuo Fan, Jian Fang, Yu Chen, Shengping Ruan

**Affiliations:** 1 College of Electronic Science & Engineering, Jilin University, Changchun 130012, China; wangql_icy@163.com (Q.W.); wangw_jlu@163.com (W.W.); fanyz97@163.com (Y.F.); fazeup@126.com (J.F.); 2 Institute of Semiconductors, Chinese Academy of Sciences, Beijing 100083, China

**Keywords:** zinc oxide, nanorods, AuND functionalization, ethyl acetate, gas sensor

## Abstract

Ethyl acetate is a critical medical indicator for detecting certain types of cancer. However, at present, available sensitive materials often exhibit drawbacks, such as high operating temperatures and poor responses to low concentrations of ethyl acetate. In this study, a ZnO nanorod sensing material was prepared using high-temperature annealing and a hydrothermally synthesized metal-organic framework (MOF) as a template. Au nanodots (AuNDs) were subsequently modified on the ZnO nanorods using an in situ ion reduction, which provided a better dispersion of Au nanodots compared with that obtained using the common reductant method. A variety of characterization methods indicate that the highly dispersed AuNDs, which possess a high catalytic activity, were loaded onto the surface as active centers, leading to a significant augmentation in the adsorption of oxygen on the surface compared with the original ZnO material. Consequently, the AuND@ZnO material exhibited heightened responsiveness to ethyl acetate at a lower operating temperature. The Au@ZnO-based sensor has a response rate (R_a_/R_g_) of 41.8 to 20 ppm ethyl acetate gas at 140 °C, marking a 17.4-fold increase compared with that of the original material. Due to its low power consumption and high responsiveness, AuND@ZnO is a promising candidate for the detection of ethyl acetate gas in medical applications.

## 1. Introduction

Ethyl acetate (EA), a common volatile organic compound pollutant, is a colorless and irritating gas with a low boiling point at atmospheric pressure. In the medical field, ethyl acetate can be detected in a patient’s exhaled breath, so it can be used as clinical diagnostic evidence for colorectal cancer and stomach cancer [1,2]. Despite being generally considered less toxic, the harm it poses to the human body should not be underestimated. Ethyl acetate can irritate the ocular conjunctiva, nasal mucosa, and upper respiratory tract. Inhalation of ethyl acetate at a certain concentration can cause acute neurological anesthesia, pulmonary edema, leukocytosis, and in severe cases, kidney and liver damage [3]. Therefore, continuous monitoring of low concentrations of ethyl acetate in specific environments (such as hospitals and factories) holds significant importance [4]. These conditions necessitate sensors with low power consumption and high responsiveness to low concentrations of EA gas. Currently, high-precision detection of such volatile organic compounds can be achieved using RFID sensors, catalytic luminescence sensors, and electrochemical sensors, among others [5,6,7].

Metal-organic frameworks (MOFs) are porous crystal materials formed by the self-assembly of metal ions or metal clusters and organic ligands through coordination bonds. They possess adjustable pore structures, functional sites, and good hydrothermal stability [8,9,10]. Moreover, the derived oxides removed from the organic ligand bonds by annealing can inherit the morphology of the original MOF material. Among them, Zn-MOF-derived oxide materials exhibit various crystal morphologies and a relatively large surface area, providing more surface reaction sites, which make them frequently employed in gas-sensing fields [11]. However, simple ZnO materials often demonstrate low gas-sensing properties, necessitating improvements through surface modification [12,13].

Noble metal nanodots have found wide applications in modifying catalytically sensitive materials owing to their excellent catalytic activity and electrical conductivity [14]. Gold nanodots (AuNDs), as the most significant member of the noble metal nanoparticle family, exhibit an electron spillover effect, significantly increasing the adsorbed oxygen ratio on the sensitive material’s surface and facilitating the rapid transfer of electrons generated in surface gas reactions to the sensitive material [15]. The in situ reduction of Au nanodots by electrostatically adsorbed materials results in a smaller size than that of noble metal nanoparticles modified using common reduction methods. The resulting size effect will affect the ZnO lattice, which can further increase the proportion of adsorbed oxygen on the material surface [16]. The excellent chemical catalytic effect of AuNDs accelerates the surface reaction of some organic gas molecules, thereby significantly enhancing the response to specific gases and reducing the optimal operating temperature of the materials [17].

In this work, Zn-MOF nanorods were synthesized by coordinating zinc ions using homophenetic acid [H_3_BTC] as the organic ligand. Ultrafine and highly dispersed gold nanodots were then synthesized on the nanorod surface via in situ reduction of HAuCl_4_ [18]. Subsequent annealing yielded Au@ZnO nanorods that exhibit high gas sensitivity properties. Characterization using XPS, TEM, and other methods revealed that the modified AuNDs on ZnO nanorods effectively increased the adsorbed oxygen ratio on the material’s surface, thereby enhancing the surface reaction rate for ethyl acetate gas. The good sensing characteristics of Au@ZnO sensors to target gas, including high responsiveness at low concentrations, low operating temperature, and admirable selectivity, can be attributed primarily to the unique electron spillover effect and good electrical conductivity of the surface AuNDs [19].

## 2. Experimental

### 2.1. Chemical Regent

Zn-MOF was synthesized using zinc nitrate hexahydrate (Zn(NO_3_)_2_·6H_2_O) and 1,3,5-benzenetricarboxylic acid (H_3_BTC), both purchased from Shanghai Aladdin Biochemical Technology Co., Ltd. (Shanghai, China). The chloroauric acid solution was prepared by dissolving chloroauric acid trihydrate (HAuCl_4_·3H_2_O) in deionized water according to a required ratio. All the solid reagents used were of analytical grade.

### 2.2. Materials Synthesis

**Figure 1:**
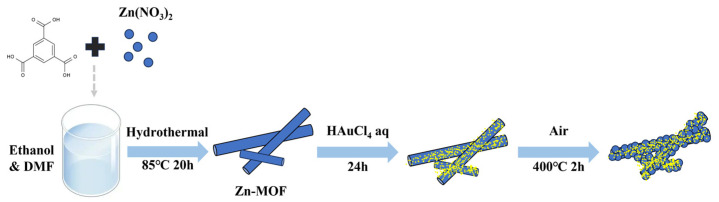
Schematic diagram of the synthesis process of AuND@ZnO nanorods.

The synthesis process is shown in Figure 1. First, Zn-MOF was prepared using a hydrothermal method [20]. Initially, 1.483 g of Zn(NO_3_)_2_·6H_2_O and 1.6 g of H_3_BTC were dissolved in 10 mL of ethanol, followed by the addition of 30 mL of DMF to the mixture. The solution was then stirred for 1 h before being transferred to a Teflon liner and heated at 85 °C for 20 h. After the hydrothermal reaction, the products were collected and cleaned three times with ethanol. Finally, the product was dried overnight in an oven at 70 °C. The resulting samples were Zn-MOF.

Subsequently, Au nanodots were modified on the Zn-MOF using an in-situ reduction method. The Zn-MOF obtained was ultrasonically dispersed in deionized water, and varying amounts of HAuCl_4_ solution, and a concentration of 20 mmol/L was added. The mixture was left for 24 h, after which the precipitate was collected. The final sample was obtained by annealing the Zn/Au-MOF in a muffle furnace at 400 °C for 2 h. The materials were named ZnAu-100, ZnAu-200, and ZnAu-400 according to the volume of HAuCl_4_ solution added (100 μL, 200 μL, and 400 μL, respectively).

### 2.3. Fabrication and Measurement of Sensors

The preparation process of the gas sensor is as follows. First, the synthesized material is mixed with anhydrous ethanol at a mass ratio of 1:4 and ground into a suspension in an agate mortar. Then, the paste is uniformly applied to a ceramic substrate with an Au measuring electrode and a Ru heating electrode, and the sensor layer is formed after the anhydrous ethanol evaporates naturally. Finally, two sets of electrodes on the ceramic substrate are welded on a hexagonal seat accordingly. The sensor is aged for two days at the aging station. Gas sensitivity is assessed using a CGS-8 analytical system (Beijing Elite, Beijing, China) at a temperature of 20 °C and relative humidity of 25%. The dynamic gas distribution system DGL-3 equipped with a humidity module is employed to introduce the measured gas into the micro gas chamber of the CGS-8 analysis system. The resistance in air is denoted as R_a_, while the resistance in the test gas is denoted as R_g_. For N-type semiconductors, the sensor’s response to reducing gases is calculated using the R_a_/R_g_ ratio, whereas that to oxidizing gases is determined using the R_g_/R_a_ ratio. Response/recovery time is defined as the time it takes for the resistance changed by 90% when the gas environment transitions.

### 2.4. Characterization

Crystal diffraction results were obtained using X-ray diffraction (XRD, Cu Kα, 1.5418 μm; D/Max 2550, Rigaku, Japan). Sample morphology information was obtained using scanning electron microscopy (SEM; JSM-7500F, JEOL, Japan), and high-resolution lattice fringes were characterized using transmission electron microscopy (TEM; JEM-2100F, JEOL, Japan). The chemical states and surface compositions of the samples were analyzed using X-ray photoelectron spectroscopy (VG ESCALAB MK II, UN).

## 3. Results and Discussion

### 3.1. Material Characterizations

**Figure 2:**
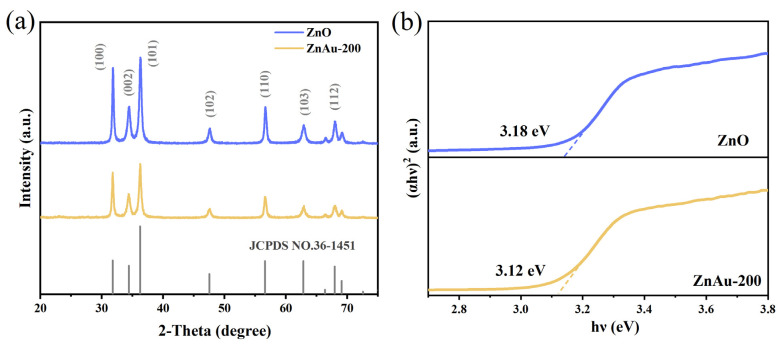
(**a**) XRD patterns of ZnAu-200 and pure ZnO materials; (**b**) Tauc diagram of ZnO and ZnAu-200.

First, XRD characterization was performed to analyze the crystal structure. The XRD results of ZnO and ZnAu-200 samples are shown in Figure 2a. All patterns correspond to the characteristics of the ZnO phase (JCPDS No. 36-1451) and match the wurtzite crystal phase well. However, due to the low Au loadings, only the ZnO phase can be identified in the XRD pattern of ZnAu-200 [21].

The Tauc diagram in Figure 2b indicates that the band gaps (E_g_) of ZnO and ZnAu-200 are 3.18 eV and 3.12 eV, respectively. A narrower E_g_ implies a smaller energy barrier for electron transfer, making it easier for electrons to be captured by oxygen molecules in air and form adsorbed oxygen, which is crucial for the detection of ethyl acetate gas. This contributes to enhanced gas-sensing performance [22].

**Figure 3:**
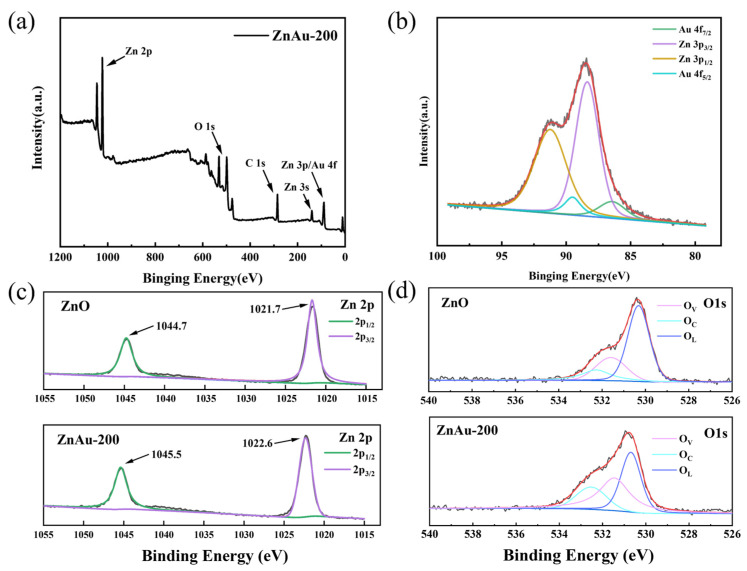
(**a**) Full XPS spectrum of ZnAu-200; (**b**) Zn 3p and Au 4f peaks resulting from the deconvolution operation; and (**c**) Zn 2p and (**d**) O 1s spectra of pure ZnO and ZnAu-200.

XPS analysis confirmed that ZnAu-200 is composed of Zn, O, C, and Au elements. The Zn 3p region overlaps with the Au 4f region, as shown in Figure 3b. The double peaks at 88.3 eV and 91.2 eV belong to Zn 3p, and the double peaks at 86.4 eV and 89.5 eV belong to Au [23]. The binding energies of the Zn 2p region are 1045.3 eV and 1022.3 eV. Compared with the control group, the Zn 2p peak in the AuZn-200 group shifted by 0.8 eV toward the low binding energy, and the relative intensity decreased [23]. According to the literature, the Fermi level of Au is lower than that of ZnO [24]. Combined with XPS, the results show that electron transfer occurs between ZnO and AuNDs on the surface of the material, and a Schottky junction is formed, which widens the material’s internal electron depletion layer and increases the baseline resistance of the material [25].

The gas-sensing property of the material is greatly affected by the proportion of oxygen adsorbed on the surface. Figure 3d shows the XPS spectrum of O 1s, which can be deconvoluted into three Gaussian peaks. The oxygen ion binding energy and relative proportions between the two materials are shown in Table 1. It can be concluded that the relative content of chemisorbed oxygen in the ZnAu-200 samples is greatly increased, which is conducive to more electron transfer on the surface of the material during the surface reaction against the target gas [26]. Thus, the resistivity of the material has a great change, which is reflected in the increase in the response value.

**Figure 4:**
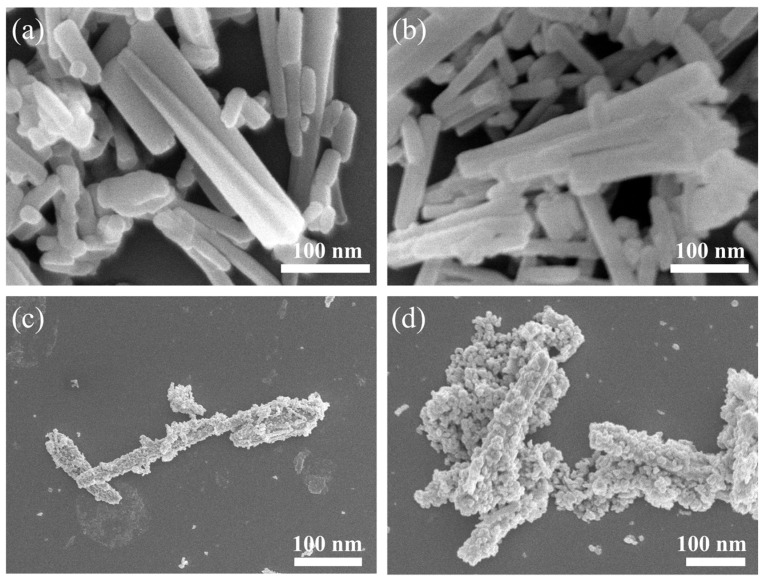
SEM images of (**a**,**b**) ZnO-MOF and (**c**,**d**) ZnAu-200.

Figure 4 illustrates the microscopic morphology of the material under a scanning electron microscope. The precursor of the sample has a typical nanorod-like structure with length of about 100 nm, which is consistent with the morphology of the ZnO-MOF precursor material, indicating that the modification with Au nanodots does not change the original metal-organic framework morphology [27]. After the organic matter is removed by calcination, the AuND@ZnO material generally inherits the stick-like morphology of the precursor material, as shown in Figure 4c. Moreover, the multi-granular changes on the surface during calcination could increase the specific surface area of the material, exposing more of the active sites of the Au dots and enhancing contact areas with the gas molecules to be measured. Moreover, the nanorod morphology enables the axial transfer of electrons, which is more conducive to the sensing reaction on the surface of the material [28].

**Figure 5:**
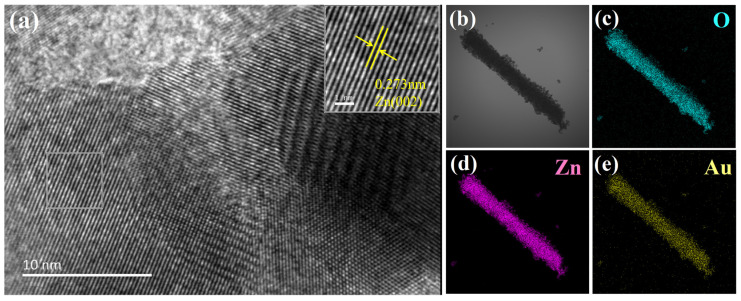
(**a**) HRTEM image and (**b**–**e**) O, Zn, and Au element mapping of ZnAu-200.

TEM image of the ZnAu-200 sample is shown in Figure 5, which is basically consistent with that in SEM images. Figure 5a show high-resolution TEM (HRTEM) images of the sample. There are two distinct sets of lattice fringes in the sample, with a spacing of 0.27 nm corresponding to ZnO (002) and 0.235 nm to Au (111) planes. The energy dispersion spectrum (EDS) is shown in Figure 5c–e, illustrating the presence and distribution of Zn, Au, and O elements. Au is uniformly distributed on the surface of the sample material in the form of nanodots.

### 3.2. Sensing Performance

**Figure 6:**
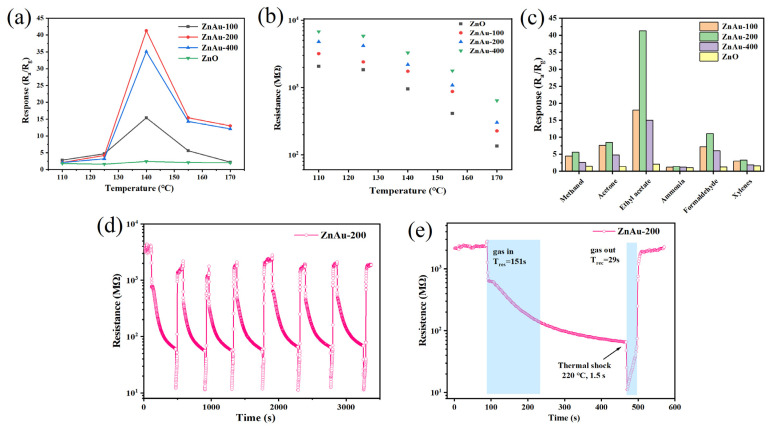
(**a**) Response–temperature relationship of sensors based on ZnAu-100, ZnAu-200, and ZnAu-400 to 20 ppm ethyl acetate; (**b**) the base resistance curve of the sensors at different operation temperatures; (**c**) responses of sensors exposed to 20 ppm of different test gases; (**d**) the repeatability test of ZnAu-200 sensor to 20 ppm ethyl acetate gas at 140 °C and (**e**) The response–recovery time curve of the ZnAu-200 sensors to 20 ppm ethyl acetate gas at 140 °C.

**Figure 7:**
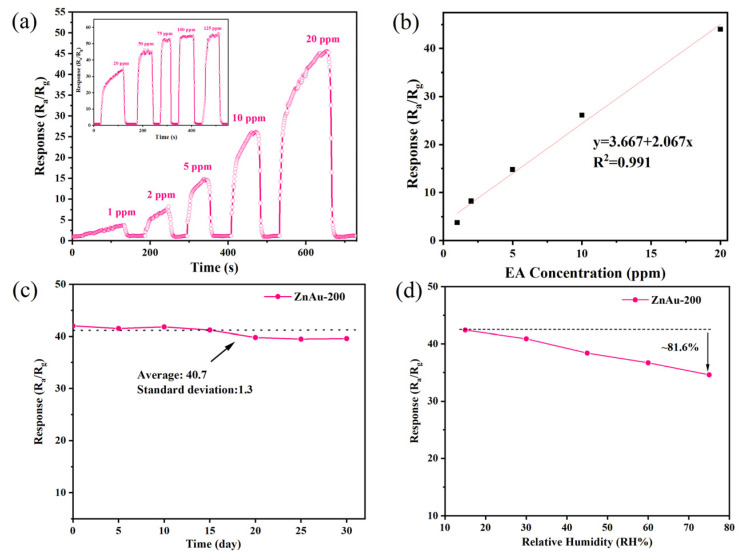
(**a**) The concentration–response curve of the ZnAu-200 sensor to 1–125 ppm ethyl acetate gas at the optimum operating temperatures; (**b**) the fitting line of ZnAu-200 sensor to 1–20 ppm; (**c**) the stability of the ZnAu-200 sensor over 30 days; (**d**) the response of the ZnAu-200 sensor to 20 ppm ethyl acetate under different relative humidity levels.

First, the optimal operating temperature of the ZnAu-200 material was determined by testing the sensor’s response to 20 ppm ethyl acetate gas at different temperatures. The response of the ZnAu-200 material to the target gas changes in a volcanic curve with the change in temperature and reaches a peak value at 140 °C, as shown in Figure 6a. A temperature that is too high or too low is not conducive to the reaction between the ethyl acetate molecule and absorbed oxygen ions, so the change in the response value of the material with temperature conforms to the change law of the gas-sensitive semiconductor material [29]. This relatively low optimal response temperature helps to reduce the power consumption of the device. The curves show that the device response value shows a trend of augmenting and then decreasing with the increasing HAuCl_4_ concentration in the modified ZnO material, reaching a maximum response value of 41.8 for the ZnAu-200 device. For comparison, the device prepared with pure ZnO has a response value of 2.4 to ethyl acetate at the same temperature. After moderate AuND modification, the response of the ZnO materials to ethyl acetate is increased by 17.4 times, which indicates that the AuND modification can improve the gas-sensitive properties of the ZnO materials. Additionally, in Figure 6b, we present the relationship between baseline resistance and temperature for pure ZnO and the three modified materials. It can be observed that as the concentration of AuCl_4_^+^ ions in the modification process increases, the baseline resistance of the materials gradually increases at the same temperature. This is due to the formation of numerous Schottky junctions between the surface-modified Au nanodots and ZnO, which deepens the electron depletion layer in ZnO, leading to a higher baseline resistance. The concentration–response relationship of the ZnAu-200 sample, which has the highest performance for ethyl acetate gas, was also tested at the optimal temperature. The dynamic resistance change curve is shown in Figure 7a. The concentration–response curve shows that the device’s response to the ethyl acetate gas in the range of 1~125 ppm can be linearly fitted. In tests of ethyl acetate gas at concentrations above 20 ppm, the response of the sensor tends to be saturated.

Selectivity is an important parameter to evaluate the performance of gas-sensing materials, so we tested the response of gas-sensing materials to 20 ppm methanol, acetone, ethyl acetate, ammonia, formaldehyde, and xylene gases at temperatures the above optimal reaction temperature (140 °C). As shown in Figure 6c, ZnO materials and ZnAu-100, ZnAu-200, and ZnAu-400 materials all show obvious selectivity for ethyl acetate, among which ZnAu-200 shows the best performance. The unique selectivity for ethyl acetate can be attributed to a synergistic effect between the release of more electrons by ethyl acetate molecules than other organic volatile gases in the surface reaction and the spillover effect of AuNDs [29].

In practical applications, different humidity levels will affect the response value of the sensor. Therefore, we tested the response of ZnAu-200 to 20 ppm ethyl acetate under different humidity conditions. It can be seen in the response–humidity curve in Figure 7d that the response of the sensor decreases with the increase in RH. This is caused by the fact that H_2_O molecules compete with the target gas for adsorbed sites on the surface at the same time. However, the material still showed an excellent response (~81.6%) at high humidity (75% RH). In terms of stability, ZnAu-200 was tested with a 20 ppm ethyl acetate cycle, as shown in Figure 6d. In order to accelerate gas desorption during the test, a 220 °C thermal pulse was used to accelerate the recovery speed. The test results show that the material has relatively stable repeatability. At the same time, a long-term stability test on the ZnAu-200 material was conducted over 30 days, as shown in Figure 7c. The results indicate that the response of the sensor decreases with increasing time, but it still shows good long-term stability.

The above performance is compared with those of the reported ethyl acetate sensors, and the results are presented in Table 2. It can be seen that the sensor based on ZnAu-200 shows a higher response at a lower operating temperature, which is conducive to reducing the power consumption of the sensing device in practical applications. Moreover, the humidity stability and response values obtained under low concentrations of the sensor in this work are obviously better than those of other sensors. Therefore, sensors based on the ZnAu-200 material can be candidates for ethyl acetate detection under a variety of conditions.

### 3.3. Sensing Mechanism

As a typical N-type semiconductor material, the conductivity of ZnO is affected by the width of the depletion layer. In different gas atmospheres, the reaction of gas molecules on the surface changes the width of the depletion layer, resulting in a change in resistance. When the sensor is set in an air environment, oxygen molecules will be adsorbed on the ZnO surface and consume electrons from the surface layer, converting them into oxygen ion species. At 100–300 °C, oxygen is mainly in the form of $O^{-}$ [31]. At the same time, since the electrons inside the material are consumed by surface oxygen, a wide electron depletion layer will be formed on the surface, and the resistance will increase, as shown in Figure 6b. When the sensor is in an ethyl acetate environment, $O^{-}$ will react with the reducing gas. The reaction can be described as follows [32]:

O_2 (gas)_ → O_2 (ads)_ (1)

O_2 (ads)_ + e^−^ → O_2_^−^ _(ads)_ (T < 100 °C) (2)

O_2_^−^ _(ads)_ + e^−^ → 2O^−^ _(ads)_ (100 °C < T < 300 °C) (3)

CH_3_COOC_2_H_5_ (g) → (CH_3_COOC_2_H_5_) _ads_ (4)

(CH_3_COOC_2_H_5_) _ads_ + 10O^−^ _(ads)_ → CO_2_ + H_2_O + 10e^−^ (5)

Oxygen species react with ethyl acetate and release electrons back to the conduction band of ZnO. This reduces the electron depletion layer width of ZnO, resulting in a decrease in sensor resistance.

Compared with pure ZnO materials, ZnO modified using an in situ reduction of AuNDs exhibit higher ethyl acetate gas-sensing properties. The improvement in the performance can be attributed to the following points. First, the Au nanodots on the nanorods help adsorb and activate more oxygen molecules and transfer them to the ZnO nanorod surface through the spillover effect [33]. This change in the proportion of oxygen molecules is reflected in the XPS test results. This chemical mechanism causes more electrons to be captured and the electron depletion layer to become wider, making the baseline resistance higher [34]. It will produce more chemisorbed oxygen and more reaction sites than original ZnO. When AuND@ZnO is exposed to ethyl acetate, more electrons will be released back to the conduction band and reduce the width of the electron depletion layer, producing a larger resistance change. Second, since the Fermi level of ZnO is higher than that of Au, electrons will be transferred from ZnO to Au nanodots at the interface between them. This forms a large number of Schottky junctions on the surface of the material, further broadening the electron depletion layer and resulting in higher baseline resistance [35,36,37].

The ion coordination-modified AuNDs formed on the surface of ZnO-MOF through the electrostatic adsorption of Au ions are smaller in size than those produced using common reduction methods [18,38]. When the Au particle size is small enough, a more obvious size effect will appear [39]. Such ultra-fine AuNDs will affect the ZnO lattice, thereby creating more surface defect oxygen and improving the gas-sensing properties of the material [40]. At the same time, the smaller the particle size of the gold nanodots is, the more obvious its chemical catalytic effect is, which is more conducive to the adsorption, diffusion, and reaction of the measured gas on the surface of the material [24,41]. AuNDs uniformly dispersed on the surface provide more reactive centers and exhibit higher response values. Excessive modification may cause the agglomeration of small-sized gold nanodots, weakening their catalytic effect on gas-sensing reactions [17]. This may explain the performance degradation of the ZnAu-400 sample. The above points together lead to the outstanding sensitivity of the Au@ZnO material to ethyl acetate gas.

## 4. Conclusions

In this study, ZnO nanorods exhibiting superior sensitivity to ethyl acetate gas were successfully prepared through in situ modification of gold nanodots on the surface of ZnO-MOF materials. Ultrafine gold nanodots uniformly distributed on ZnO nanorods play a crucial role in enhancing the gas-sensing properties. The electron spillover effect and catalysis facilitated by AuNDs on the surface of ZnO nanorods provide additional reaction centers to promote the surface reaction of ethyl acetate molecules. In situ reduction-modified ultrafine gold nanodots have an effect on the ZnO lattice, which in turn increases the adsorbed oxygen on the nanomaterial’s surface. The above mechanisms collectively lead to the improvement in the gas-sensing properties of the materials. The test results demonstrate that the response of the pristine ZnO materials to 20 ppm ethyl acetate is increased by 17.4 times, reaching 41.8. Hence, AuND@ZnO nanorods can be used as a superior gas-sensitive material for ethyl acetate monitoring.

## Figures and Tables

**Table 1:** XPS spectra fitting results of oxygen species in ZnO and ZnAu-200.

Sample	Oxygen Species	Binding Energy (eV)	Relative Percentage (%)
ZnO	O_C_ (chemisorbed)	532.2	15.72%
	O_V_ (vacancy)	531.5	24.19%
	O_L_ (lattice)	530.3	60.08%
ZnAu-200	O_C_ (chemisorbed)	532.5	19.42%
	O_V_ (vacancy)	531.4	42.56%
	O_L_ (lattice)	530.6	38.00%

**Table 2:** Comparison of ethyl acetate sensing results from the previous literature and this study.

Materials	OT (°C)	Conc (ppm)	Res (R_a_/R_g_)	LOD	Refs.
PrFeO_3_/α-Fe_2_O_3_ octahedrons	206	20	11	1 ppm	[29]
CoWO_4_/α-Fe_2_O_3_ octahedrons	206	100	24.1	1 ppm	[30]
Flower-like Au-ZnO	240	20	0.5	10 ppm	[13]
NiFe_2_O_4_ nanoboxes	120	20	12	1 ppm	[3]
Au@ZnO nanorods	140	20	41.8	1 ppm	This work

## Data Availability

The authors confirm that the data supporting the findings of this study are available within the article and Supplementary Materials.

## Supplementary Materials

The following supporting information can be downloaded at: https://www.mdpi.com/article/10.3390/s24216931/s1.
