# Supplementary figures and images for: Functionalization of ZnO Nanorods with Au Nanodots via In Situ Reduction for High-Performance Detection of Ethyl Acetate
Source: Sensors (Basel). 2024 Oct 29;24(21):6931. doi: 10.3390/s24216931 (PMC11548670; doi:10.3390/s24216931)

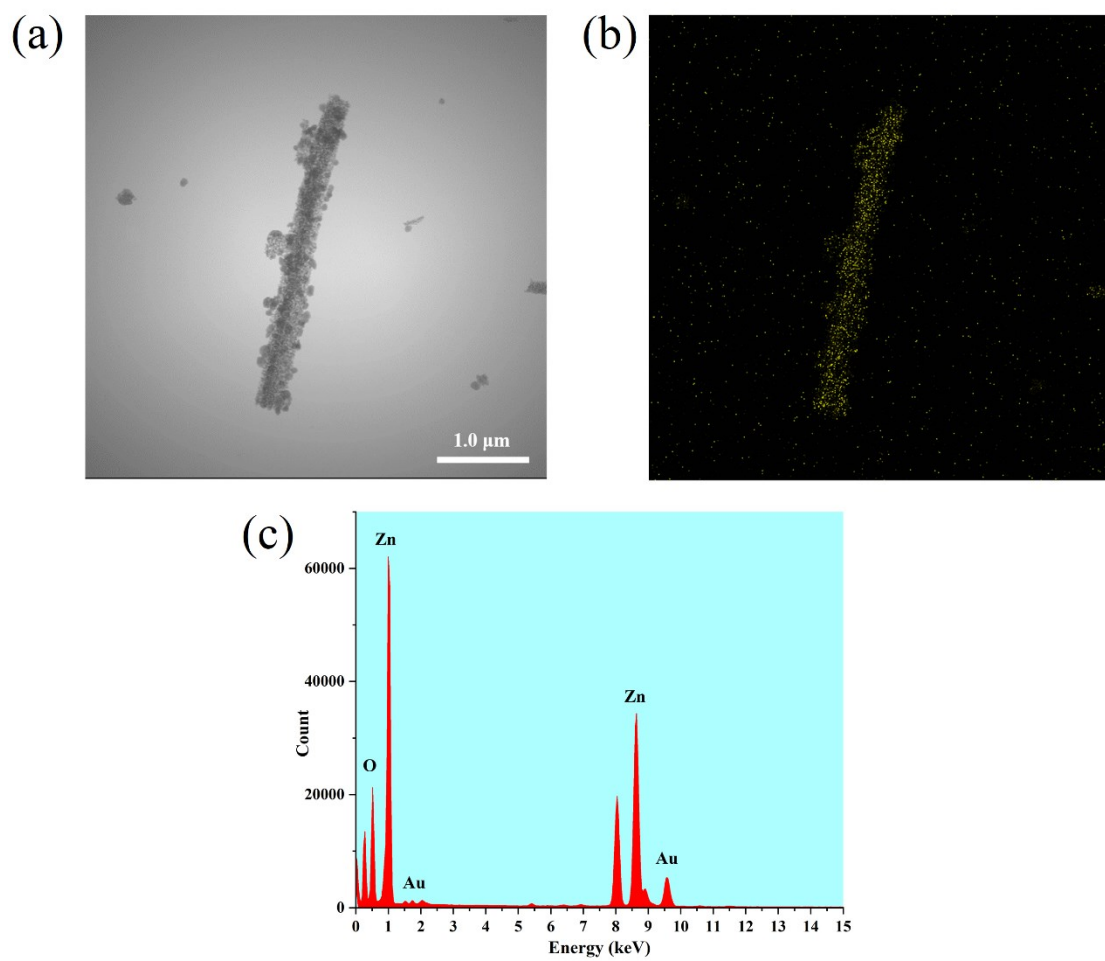

**Figure S1.** (a) TEM image of ZnAu-200 nanorod; (b) Au elemental mapping of ZnAu-200 nanorod; (c) the EDS spectrum.

Supplement: Supplementary file 1 [file sensors-24-06931-s001.zip › sensors-3248744-supplementary.pdf]
